# Change in decay rates of dioxin-like compounds in *Yusho* patients

**DOI:** 10.1186/s12940-016-0178-0

**Published:** 2016-09-07

**Authors:** Shinya Matsumoto, Manabu Akahane, Yoshiyuki Kanagawa, Jumboku Kajiwara, Chikage Mitoma, Hiroshi Uchi, Masutaka Furue, Tomoaki Imamura

**Affiliations:** 1 Department of Public Health, Health Management and Policy, Nara Medical University School of Medicine, 840 Shijo-cho, Kashihara, Nara 634-8521 Japan; 2 Fukuoka Institute of Health and Environmental Sciences, 39 Mukaizano, Dazaifu, Fukuoka Japan; 3 Department of Dermatology, Graduate School of Medical Sciences, Kyushu University, 3-1-1 Maidashi, , Higashi-ku Fukuoka, Japan

**Keywords:** *Yusho*, 2,3,4,7,8-pentachlorodibenzofuran (PeCDF), Half-life, Dioxin, Dioxin-like compounds, Aging

## Abstract

**Background:**

Once ingested, dioxins and dioxin-like compounds are excreted extremely slowly. Excretion can be evaluated by its half-life. Half-lives estimated from observed concentrations are affected by excretion and ongoing exposure. We investigated the change in apparent half-life using a theoretical model based on exposure to dioxin and dioxin-like compounds.

**Methods:**

We carried out longitudinal measurements of the blood concentration of dioxins and dioxin-like compounds in a *Yusho* cohort during 2002 to 2010. We estimated the change in decay rates of 2,3,4,7,8-PeCDF and octachlorodibenzodioxin (OCDD) using a second-order equation.

**Results:**

We found that the decay rate of OCDD increased, whereas the decay rate of 2,3,4,7,8-PeCDF of patients with a relatively high concentration of 2,3,4,7,8-PeCDF decreased. OCDD results were in accordance with decreasing levels of dioxin and dioxin-like compounds in the environment. The decay rate of OCDD in the body was affected by the decay rate of OCDD in the environment by ingestion because it was near the steady-state. In contrast, the decay rate of 2,3,4,7,8-PeCDF in the body was affected less by ingestion from the environment because it was far higher than in the steady-state.

**Conclusion:**

We demonstrated that the level of 2,3,4,7,8-PeCDF in the environment is decreasing. The excretion half-life is longer than the environmental half-life, thus the excretion half-life in a *Yusho* patient is increased.

## Background

*Yusho* refers to a mass food poisoning that occurred in western Japan in 1968. Early studies indicated that *Yusho* was caused by polychlorinated biphenyls (PCBs). According to a number of subsequent studies, though, it is now accepted that 2,3,4,7,8-pentachlorodibenzofuran (PeCDF) was the main causative compound of *Yusho* [1, 2]. The concentrations of dioxins and dioxin-like compounds in the blood of *Yusho* patients have been measured at annual medical checkups since 2001 [3, 4].

Once ingested, dioxins and dioxin-like compounds are excreted extremely slowly. Given their health implications, there has been a great deal of interest in the half-lives of these compounds in humans. In patients with high blood concentrations of dioxin-like compounds, half-lives of 1.1 years have been reported, increasing to 7.2 years in patients with low blood concentrations [5]. Other estimates on half-lives of dioxin-like compound include 8.9 years by Masuda et al. [6], 9.6 years by Ryan et al. [7] and 9.1 years by Iida et al. [8]. Many researchers have reported half-lives of PCBs to be less than 10 to 15 years [9, 10]. Among patients with blood concentrations of 2,3,4,7,8-pentachlorodibenzofuran (2,3,4,7,8-PeCDF) ≥50 pg/g lipid, we identified two groups: one showing an apparent half-life of ≈ 10 years and the other showing no reduction in 2,3,4,7,8-PeCDF levels over time [11]. This suggests that the latter group of patients maintained high blood levels of 2,3,4,7,8-PeCDF.

Since the medical checkups began, the group having a 2,3,4,7,8-PeCDF apparent half-life of around 10 years became smaller while the group having a near infinite apparent half-life became larger [12]. Therefore, the excretion half-life changed in individual patients. Milbrath et al. [13] pointed out that excretion half-life was affected by menopause, and other researchers reported that those changes in apparent half-lives were affected by intake [14]. In this paper we evaluate the changes in apparent half-life of dioxin-like compounds.

## Methods

The subjects were 354 patients whose blood concentration of 2,3,4,7,8-PeCDF had been measured three or more times at annual *Yusho* medical checkups between 2002 and 2010, i.e., 34–42 years since exposure, and for whom the period from the first to last measurement was over 4 years. We examined two chemicals: 2,3,4,7,8-PeCDF, which is the causative chemical [2], and octachlorodibenzodioxin (OCDD), which is a chemical found in *Yusho* patients but less commonly in the general public [15]. Patient distribution according to 2,3,4,7,8-PeCDF concentration in 2006 (middle of the observation period) is shown in Table 1. This research was approved by Nara Medical University Ethics Committee (No. 281–2).

**Table 1:** Distribution of 2,3,4,7,8-PeCDF concentration

Concentration range (pg/g lipid)	Number of patients
0–10	43
10–20	78
20–50	77
50–100	34
>100	122

The excretion of dioxins and dioxin-like compounds is proportional to body burden. If there is no intake of these compounds and their quantity decreases in proportion to body burden then their decay will be logarithmic. However, body burden of dioxins and dioxin-like compounds is affected by intake as well as excretion. Therefore, the body burden itself will not decay logarithmically.

**Fig. 1:**
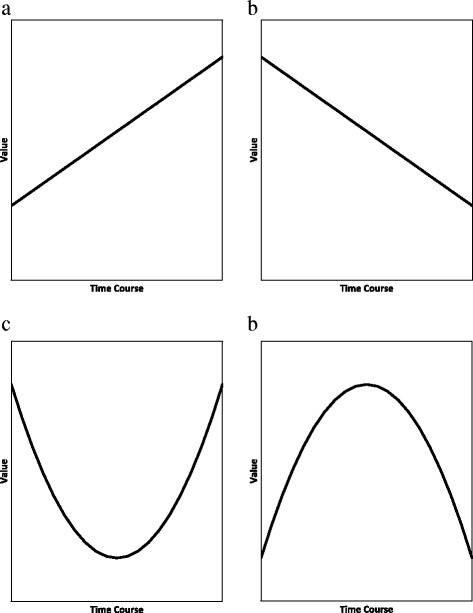
Linear and parabolic curves. **a** First derivative is positive and second derivative is zero; (**b**) first derivative is negative and second derivative is zero; (**c**) second derivative is positive; (**d**) second derivative is negative

Time course curves can be characterized by second-order differentiation. Figure 1(a) shows a linear curve with a positive gradient; the first derivative is positive and the second derivative is zero. Likewise, Fig. 1(b) shows a linearly decreasing curve with negative first derivative and zero second derivative. Curves with non-zero second derivatives are shown in Fig. 1(c) and (d); the former curve is concave with positive second derivative, and the latter is convex with negative second derivative. The rate of change in body burden is expressed by its second-order derivative with respect to time.

For the logarithm of body burden, which was calculated from concentration and weight, we examined the second derivative by second-order regression. The body burden *C*_*it*_ of patient *i* at time *t* is given by:

1 $$ \log {C}_{it}={\beta}_i+{\alpha}_1t+{\alpha}_2{t}^2+{\gamma}_1{w}_{1 it}+{\gamma}_2{w}_{2 it} $$

where *t* is the time, with *t* = 0 denoting 2006 (middle of the observation period), *α*_*2*_ is the coefficient for a second-order derivative, *α*_*1*_ is the first-order derivative at time *t* = 0, *β*_*i*_ is the reference value for each individual, *W*_*1it*_ is the weight and *W*_*2it*_ is the lipid in blood (%) of patient *i* at time *t*, and *γ*_*j*_ is the coefficient for weight and lipid in blood. Aylward et al. used an estimate of body fat as volume of distribution [16], but we used weight and lipid in blood.

We combined all observed concentrations into equation (2):

2 $$ y=X\cdot \beta +T\cdot \alpha +W\cdot \gamma +\varepsilon $$

where y is the vector of the logarithm of dioxins and dioxin-like compounds in blood lipid concentration, *Xβ* is the base concentration for each patient, *Tα* is the change in time, *Wγ* is an adjustment term according to weight and lipid in blood, and *ε* is an observation error. *X* is a matrix with the total number of measurements rows and number of patient columns. *T* is a matrix with the total number of measurement rows and two columns, second-order and first-order for time. *W* is the matrix with the total number of measurement rows and two columns, weight and lipid. Equation (3) is a matrix form:

3 $$ \mathrm{y}=\left(\begin{array}{ccc}\hfill X\hfill & \hfill T\hfill & \hfill W\hfill \end{array}\right)\left(\begin{array}{c}\hfill \beta \hfill \\ {}\hfill \alpha \hfill \\ {}\hfill \gamma \hfill \end{array}\right)+\upvarepsilon $$

By solving multiple linear regressions with the lm function in R, we could estimate first- and second-order derivatives. It was assumed that patients in the same group had same trend with regard to time. Conversely, it was assumed that patients in different groups had a different trend with respect to time. For the case of one patient and no second-order coefficient measurements, equation (3) equals leads to an equation for estimation of decay rate:

4 $$ {k}_a=\frac{ \ln {C}_{t2}- \ln {C}_{t1}}{\varDelta t} $$

The governing equation for a one-compartment PK model having a constant intake and an excretion proportional to body burden is

5 $$ \frac{dQ}{dt}=-\lambda \cdot Q+I $$

By integrating Eq. (5) we obtain the body burden as a function of time,

6 $$ Q(t)=\left(\frac{e^{\lambda t}\cdot I}{\lambda }+{Q}_0\right)\cdot {e}^{-\lambda t} $$

*Q*_0_ is a constant of integration corresponding to time *t* = 0. Taking the derivative of the logarithm of Eq. (6) gives

7 $$ \frac{d}{dt} \log Q(t)=\frac{1}{Q(t)}\frac{d}{dt}Q(t)=-\frac{1}{Q(t)}\left(\lambda \cdot Q-I\right) $$

and taking a second derivative gives

8 $$ \frac{d^2}{d{t}^2} \log Q(t)=-\frac{1}{Q^2}\cdot {\left(\frac{dQ}{dt}\right)}^2+\frac{1}{Q}\cdot \frac{d^2Q}{d{t}^2}=\frac{1}{Q^2}\cdot \left(\lambda \cdot Q-I\right) $$

The first-order and second-order equations have opposite signs,

9 $$ \frac{d^2}{d{t}^2} \log Q(t)=-\frac{1}{Q}\cdot \frac{d}{dt} \log Q(t) $$

We estimated the first- and second-order coefficients, equivalent to the decay rate and change in decay rate, for OCDD and 2,3,4,7,8-PeCDF for each patient group shown in Table 1.

## Results

**Fig. 2:**
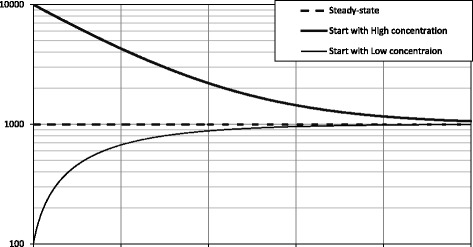
Change in body burden in the constant-intake model

Figure 2 shows the time progression of body burden with a constant intake of dioxins for initially high and low concentrations, governed by Eq. (5). The dashed line corresponds to the steady-state of the body burden being equal to the integration constant, *Q*(*t*) = *Q*_0_. In a patient who underwent accidental exposure to a high level of dioxins, the body burden will decay exponentially as the first derivative is negative. In a patient who has a lower body burden than the steady-state level, the body burden will approach the steady-state level in an exponential fashion; i.e., the first derivative is positive and the second derivative is negative. Bartell evaluated how intake affects half-life if intake is constant [14].

**Fig. 3:**
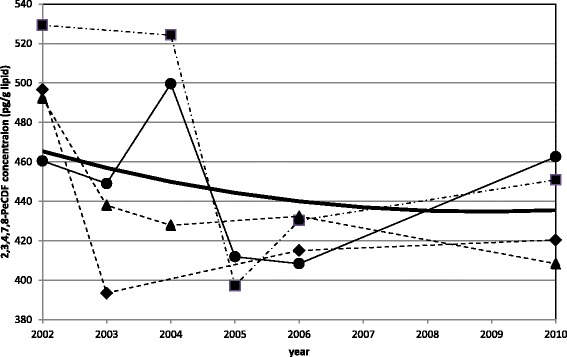
Trend for 4 typical patients and estimation of a curve for a high concentration

Table 2 shows the coefficients and *p*-values for 2,3,4,7,8-PeCDF body burden. The second-order coefficients for patients who have 2,3,4,7,8-PeCDF concentrations greater than 50 pg/g lipid are positive; i.e., the curves are concave. For patients who have less than 50 pg/g lipid, the *p*-values are higher than 5 %. The coefficient for second-order derivative is not determined, i.e., the curve is linear. The first-order coefficients are negative for patient concentrations greater than 50 pg/g lipid. For concentrations <50 pg/g lipid, *p* > 0.05 (i.e., was not significant), so the coefficient for the first-order derivative would not change the body burden. Only the group with a lipid concentration >50 pg/g had a negative apparent rate of change in concentration. Figure 3 shows the estimated time trend curve and typical changes for 4 patients with a high concentration of 2,3,4,7,8-PeCDF.

**Table 2:** Apparent elimination rate and change in elimination rate for body burden of 2,3,4,7,8-PeCDF

Concentration range (pg/g lipid)	Second-order (equivalent to minus change in apparent elimination rate in 2006)		First-order (equivalent to minus apparent elimination rate)	
	coefficient	*p*-value	coefficient	*p*-value
0–10	−0.000596	0.599665	−0.004032	0.165814
10–20	0.000464	0.463854	−0.000462	0.786707
20–50	0.000643	0.182574	0.001669	0.229181
50–100	0.002124	7.99 × 10^−5^	−0.005452	0.000216
>100	0.001436	9.68 × 10^−7^	−0.008294	<2 × 10^−16^

Table 3 presents the coefficients and *p*-values for OCDD body burden. All second-order coefficients for all concentration groups were negative and *p* < 0.05 %, so the curves were convex. First-order coefficients were negative and *p* < 0.05 % for all concentrations groups, so the change in OCDD concentration was negative (i.e., OCDD concentration was declining).

**Table 3:** Apparent elimination rate and change in elimination rate for body burden of OCDD

Concentration range (pg/g lipid)	Second-order (equivalent to minus change in apparent elimination rate in 2006)		First-order (equivalent to minus apparent elimination rate)	
	coefficient	*p*-value	coefficient	*p*-value
0–10	−0.006366	4.80 × 10^−7^	−0.014329	7.87 × 10^−6^
10–20	−0.003289	5.81 × 10^−5^	−0.019541	<2 × 10^−16^
20–50	−0.001897	0.006100	−0.020541	<2 × 10^−16^
50–100	−0.002991	0.003593	−0.012243	2.04 × 10^−5^
>100	−0.004341	<2 × 10^−16^	−0.009034	2.94 × 10^−9^

## Discussion

In Japan, young people have lower concentrations of 2,3,4,7,8-PeCDF than older people [17, 18]. The model in which the environmental concentration is decreasing is the more realistic model. If intake is constant, older people may have a higher concentration than that in young people due to accumulation of dioxin and dioxin-like compounds. However, in this model, if the concentration is near steady-state, the rate of increase is slowed down. If the concentration in the environment is decreasing, body burden is decreased because of a total decrease in exposure from birth. The production of dioxins was restricted in the 1970s and concentrations of dioxins and dioxin-like compounds in the environment subsequently fell. Thus, the intake of these compounds is no longer constant. Some scholars have reported that levels of dioxins and dioxin-like compounds in the environment have decreased [10, 19, 20].

Let us assume that the intake is decreasing according to the following equation,

10 $$ I(t)={I}_0\cdot {e}^{-{\lambda}_0\cdot t} $$

We combine Eq. (5) and Eq. (10) to get

11 $$ \frac{dQ}{dt}=-\lambda \cdot Q+{I}_0\cdot {e}^{-{\lambda}_0\cdot t} $$

Integrating Eq. (11) gives body burden as a function of time,

12 $$ Q(t)=\left(\frac{I_0}{\lambda -{\lambda}_0}\cdot {e}^{\left(\lambda -{\lambda}_0\right)\cdot t}+{Q}_0\right)\cdot {e}^{-\lambda \cdot t} $$

**Fig. 4:**
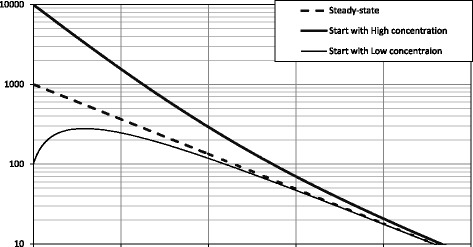
Change in body burden in the decreasing intake model

Figure 4 plots body burden with a decreasing intake for patients with initially high and low concentrations and a patient at steady-state. In the patient with initially high concentration, the body burden decays exponentially at an ever-slower rate. Thus, the second derivative is positive. In the patient with initially low concentration, the body burden increases then decreases, which corresponds to polarity changes in the first derivative and negative second derivative throughout.

2,3,4,7,8-PeCDF is a causative compound of *Yusho. Yusho* patients have a higher concentration of 2,3,4,7,8-PeCDF than do the general public. *Yusho* patients have a lower concentration of OCDD than do the general public [15].

In the OCDD results in Table 3, the first- and second-order differentiation coefficients are negative. The OCDD concentration was a convex curve. *Yusho* patients had a lower concentration of OCDD than do the general public, and the body burden approached the steady-state. If the body burden was approaching but not yet at steady-state and the OCDD environmental level was reducing faster than the body excretion rate, then the first and second derivatives would be negative. Ritter et al. [19] estimated the excretion half-life of dioxins from measurements of people at one time point using a model that assumed a decreasing level of dioxins in the environment. These findings are not in accordance with the constant-intake model. OCDD data were in accordance with the hypothesis of decreasing levels of dioxins and dioxin-like compounds in the environment.

Results for 2,3,4,7,8-PeCDF summarized in Table 2 are consistent with those shown in Fig. 2 for a patient group having >50 pg/g lipid; second derivatives were positive and the first derivatives were negative, and a concave curve was produced. In the group having less than 50 pg/g lipid, the *p*-values are higher than 5 %, and the sign of the coefficients cannot not be determined because body burden is low, intake and excretion have similar values and there are substantial differences between patients.

Therefore, to accurately predict body burden, a model should assume a decreasing level of 2,3,4,7,8-PeCDF. The decrease in intake will be influenced directly. If decay in the environment occurs at a constant rate and the environment half-life is longer than the excretion half-life, then the apparent half-life converges to the environment half-life [21]. The variation of ingestion does not affect the converged reduction rate. In the 2,3,4,7,8-PeCDF results, however, there is no decrease in the concentration for patients having less than 50 pg/g lipid. If the environment half-life is shorter than the excretion half-life, then the apparent half-life converges to the excretion half-life, and the second derivative is negative. This hypothesis is not in accordance with 2,3,4,7,8-PeCDF results.

We reported that concentrations of 2,3,4,7,8-PeCDF in *Yusho* patients are decreasing very slowly and prolonging the apparent half-life [12]. With a decreasing concentration in the environment and a constant excretion half-life, the apparent half-life of high concentrations is shortening. Our report is inconsistent with the constant excretion model. The prolongation of the apparent half-lives of 2,3,4,7,8-PeCDF at high concentrations is caused by the prolonging of excretion half-lives.

## Conclusions

If a person is exposed to high levels of dioxins and dioxin-like compounds in the environment and if excretion half-life is shorter than the environmental half-life, then the apparent half-life will be more profoundly influenced by to the environmental half-life. Conversely, if the excretion half-life is longer than the environmental half-life, then the apparent half-life will be preferentially influenced by the excretion half-life. We demonstrated that the level of 2,3,4,7,8-PeCDF in the environment is decreasing. Our results show that the excretion half-life is longer than the environmental half-life, thus the excretion half-life in a *Yusho* patient is increasing.

## Abbreviations

2,3,4,7,8-PeCDF: 2,3,4,7,8-pentachlorodibenzofuran
OCDD: octachlorodibenzodioxin
PCB: polychlorinated biphenyl
PeCDF: pentachlorodibenzofuran
