# Erratum to: Change in decay rates of dioxin-like compounds in *Yusho* patients

**DOI:** 10.1186/s12940-016-0192-2

**Published:** 2016-11-16

**Authors:** Shinya Matsumoto, Manabu Akahane, Yoshiyuki Kanagawa, Jumboku Kajiwara, Chikage Mitoma, Hiroshi Uchi, Masutaka Furue, Tomoaki Imamura

**Affiliations:** 1 Department of Public Health, Health Management and Policy, Nara Medical University School of Medicine, 840 Shijo-cho, Kashihara, Nara 634-8521 Japan; 2 Fukuoka Institute of Health and Environmental Sciences, 39 Mukaizano, Dazaifu, Fukuoka Japan; 3 Department of Dermatology, Graduate School of Medical Sciences, Kyushu University, 3-1-1 Maidashi, Higashi-ku, Fukuoka Japan

## Erratum

*n.b. The error described below was the result of a technical error, and thus was* ***not*** *the fault of the authors*.

This article [1] contains an error characterised by a discrepancy in the presentation of equation (6) between the article’s HTML and PDF formats.

The version of the equation present in the HTML is correct, while conversely the version present in the PDF is incorrect.

As such, the correctly formatted equation is as follows:

6 $$ Q(t)=\left(\frac{e^{\lambda t}\cdot I}{\lambda }+{Q}_0\right)\cdot {e}^{-\lambda t} $$

## References

[1] Matsumoto S (2016). Change in decay rates of dioxin-like compounds in *Yusho* patients. Environ Health.

